# HBV Enhances Sorafenib Resistance in Hepatocellular Carcinoma by Reducing Ferroptosis via SRSF2-Mediated Abnormal PCLAF Splicing

**DOI:** 10.3390/ijms24043263

**Published:** 2023-02-07

**Authors:** Lijuan Liu, Zhao Lv, Miao Wang, Dongyan Zhang, Dongying Liu, Fan Zhu

**Affiliations:** State Key Laboratory of Virology and Hubei Province Key Laboratory of Allergy and Immunology, Department of Medical Microbiology, School of Basic Medical Sciences, Wuhan University, Wuhan 430071, China

**Keywords:** hepatitis B virus (HBV), hepatocellular carcinoma (HCC), alternative splicing, proliferating cell nuclear antigen clamp-associated factor (PCLAF), sorafenib resistance, ferroptosis

## Abstract

Hepatocellular carcinoma (HCC) is one of the most lethal human cancers. Hepatitis B virus (HBV) infection accounts for nearly 50% of HCC cases. Recent studies indicate that HBV infection induces resistance to sorafenib, the first-line systemic treatment for advanced HCC for more than a decade, from 2007 to 2020. Our previous research shows that variant 1 (tv1) of proliferating cell nuclear antigen clamp-associated factor (PCLAF), overexpressed in HCC, protects against doxorubicin-induced apoptosis. However, there are no reports on the relevance of PCLAF in sorafenib resistance in HBV-related HCC. In this article, we found that PCLAF levels were higher in HBV-related HCC than in non-virus-related HCC using bioinformatics analysis. Immunohistochemistry (IHC) staining of clinical samples and the splicing reporter minigene assay using HCC cells revealed that PCLAF tv1 was elevated by HBV. Furthermore, HBV promoted the splicing of PCLAF tv1 by downregulating serine/arginine-rich splicing factor 2 (SRSF2), which hindered the inclusion of PCLAF exon 3 through a putative *cis*-element (116–123), “*GATTCCTG*”. The CCK-8 assay showed that HBV decreased cell susceptibility to sorafenib through SRSF2/PCLAF tv1. HBV reduced ferroptosis by decreasing intracellular Fe^2+^ levels and activating GPX4 expression via the SRSF2/PCLAF tv1 axis, according to a mechanism study. Suppressed ferroptosis, on the other hand, contributed to HBV-mediated sorafenib resistance through SRSF2/PCLAF tv1. These data suggested that HBV regulated PCLAF abnormal alternative splicing by suppressing SRSF2. HBV caused sorafenib resistance by reducing ferroptosis via the SRSF2/PCLAF tv1 axis. As a result, the SRSF2/PCLAF tv1 axis may be a prospective molecular therapeutic target in HBV-related HCC, as well as a predictor of sorafenib resistance. The inhibition of the SRSF2/PCLAF tv1 axis may be crucial in the emergence of systemic chemotherapy resistance in HBV-associated HCC.

## 1. Introduction

In 2020, primary liver cancer is expected to be the sixth most frequent disease and the third main cause of cancer death worldwide, with an estimated 906,000 new cases and 830,000 deaths [1]. Hepatocellular carcinoma (HCC) accounts for 75% to 85% of all primary liver cancers [1]. Hepatitis B virus (HBV) is responsible for around two out of every three instances of liver cancer in less developed countries, but only one out of every four cases in more developed countries [2]. HBV has emerged as a major threat to HCC, maybe because of chemoresistance, a common issue in the treatment of HCC [3]. Sorafenib, a multi-kinase inhibitor, has been a successful first-line targeted therapy for late-stage HCC for more than ten years, from 2007 to 2020 [4]. HBV components, including large HBs, HBx, and HBc, can contribute to sorafenib resistance in HCC [5,6,7]. However, the mechanism by which HBV participates in sorafenib resistance requires additional investigation.

Alternative splicing is a critical process for gene expression control and proteome diversity [8]. Aberrant alternative splicing events are common throughout the development of HCC. Chemoresistance can be exacerbated by oncogenic variants caused by splicing [9,10]. Proliferating cell nuclear antigen clamp-associated factor (PCLAF) (Gene ID: 9768) regulates DNA replication, DNA repair, cell cycle progression, and cell proliferation [11,12,13], and has been linked to the development of a number of cancers [13,14,15,16]. It has two variants caused by exon 3 inclusion (PCLAF tv1) or exon 3 skipping (PCLAF tv2). Previously, we discovered that PCLAF tv1 performed an oncogenic role in late-stage HCC and caused doxorubicin resistance [17], but PCLAF tv2 reduced PCLAF tv1′s oncogenic effect [18]. In HCC, HBV has been implicated in the abnormal alternative splicing of host oncogenes [19,20]. HepG2.2.15, a cell line derived from HepG2 by HBV DNA integration [21], has higher PCLAF tv1 protein levels than HepG2 [17], showing that HBV may promote PCLAF tv1 splicing. Alternative splicing is regulated by *cis*-elements cooperating with *trans*-acting factors. *Cis*-elements are short sequences located near or within an alternatively spliced exon. Serine-arginine (SR) proteins are important *trans*-acting factors [8,22]. SRSF2, a 35 KDa SR protein, is involved in both exon inclusion and exclusion [23,24]. The involvement of SRSF2 in HCC is obscure. A recent study discovered that HBV downregulated SRSF2 and that it was involved in HBV gene expression regulation [25]. It is speculated that SRSF2 is implicated in HBV-associated HCC. However, whether HBV regulates PCLAF splicing via SRSF2 and the role of PCLAF in HBV-induced sorafenib resistance are unknown.

Ferroptosis, a type of iron-dependent programmed cell death caused by excessive lipid peroxidation, was postulated by Dixon et al. in 2012 [26]. Inhibiting cell membrane translocators such as cystine/glutamate translocators (also known as system X_c_^−^) or activating transferrin, as well as suppressing intracellular antioxidant enzymes such as glutathione peroxidase 4 (GPX4), causes ferroptosis [26]. Sorafenib induces ferroptosis by blocking system X_c_^−^ absorption of cystine [27,28]. Deferoxamine, an iron chelator, dramatically lowers sorafenib sensitivity on Huh7, an HCC cell line, suggesting that sorafenib has anticancer efficacy via triggering ferroptosis in HCC [27,28]. However, more research is needed to determine whether HBV is implicated in sorafenib resistance via ferroptosis.

In this study, PCLAF tv1 was discovered to be the predominant variant in HBV-positive HCC tissues and cells. HBV-induced sorafenib resistance was achieved by increasing PCLAF tv1 splicing with reducing SRSF2 expression. Through a suspected *cis*-element on PCLAF Ex3 (116–123), “*GATTCCTG*”, SRSF2 hampered the inclusion of PCLAF exon 3. HBV was found to inhibit ferroptosis by reducing intracellular Fe^2+^ levels and increasing GPX4 expression via the SRSF2/PCLAF tv1 axis. Conversely, suppressed ferroptosis contributed to HBV-mediated sorafenib resistance through the SRSF2/PCLAF tv1 axis. Inhibiting the SRSF2/PCLAF tv1 axis could be important in the development of systemic chemotherapy resistance in HBV-associated HCC.

## 2. Results

### 2.1. HBV Promotes Sorafenib Resistance in HCC by Increasing PCLAF tv1 Splicing

**Figure 1:**
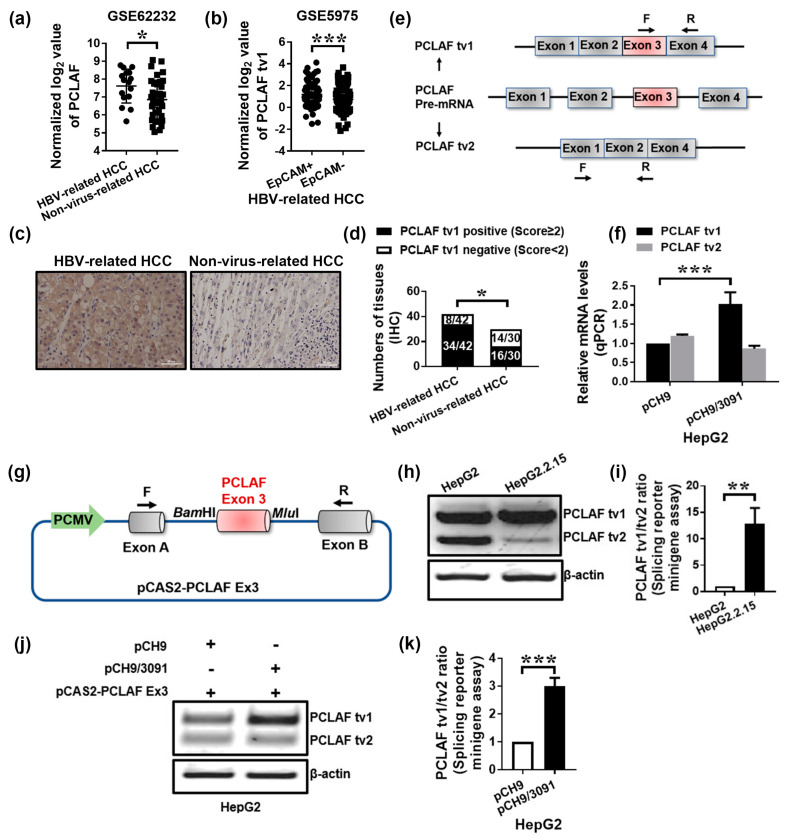
HBV increases PCLAF tv1 splicing in HCC cells. The normalized log_2_ expression of PCLAF in GSE62232 was shown. * *p* < 0.05 (**a**). The normalized log_2_ value of PCLAF tv1 in GSE5975 was presented. *** *p* < 0.001 (**b**). PCLAF tv1 staining in HBV-related HCC and non-virus-related HCC was detected using immunohistochemistry. The images are representative (scale bar, 50 μm) (**c**). The graph displays the ratio of PCLAF tv1-positive to -negative in HBV-related HCC and non-virus-related HCC using immunohistochemistry. * *p* < 0.05 (**d**). Alternative splicing in human PCLAF pre-mRNA exon 3 resulted in the production of PCLAF tv1 and tv2 mRNAs. Furthermore, the primers specific for PCLAF tv1 and tv2 used in qPCR were identified (**e**). PCLAF tv1 and tv2 mRNA levels were measured in HepG2 cells transfected with pCH9 or pCH9/3091 by qPCR. *** *p* < 0.001 (**f**). The diagram depicts the assembly of the pCAS2-PCLAF Ex3 minigene. The PCLAF wildtype exon 3 and surrounding intronic sequences were inserted into the *Bam*HI and *Mlu*I restriction sites of the pCAS2 vector, which contained a two-exon splicing reporter minigene (**g**). Following transient transfection of the pCAS2-PCLAF Ex3 minigene in HepG2 and HepG2.2.15 cells, exogenous PCLAF tv1 and tv2 levels were measured using a splicing reporter minigene assay (**h**). The graph shows the tv1/tv2 ratio of exogenous PCLAF in HepG2 and HepG2.2.15 cells. ** *p* < 0.005 (**i**). Following transient transfection of the pCAS2-PCLAF Ex3 minigene in HepG2 cells transfected with pCH9 or pCH9/3091 plasmids, exogenous PCLAF tv1 and tv2 levels were assessed using a splicing reporter minigene assay (**j**). The graph shows the exogenous PCLAF tv1/tv2 ratio in pCH9 or pCH9/3091 plasmid transfected HepG2 cells. *** *p* < 0.001 (**k**).

The Gene Expression Omnibus (GEO) Dataset GSE62232, which contains 16 HBV-related HCC specimens and 37 non-virus-related HCC specimens [29], revealed that PCLAF levels were significantly higher in HBV-related HCC tissues than in non-virus-related HCC tissues (*p* < 0.05; Figure 1a), indicating that HBV upregulated PCLAF. PCLAF tv1 was found to be more abundant in EpCAM-positive HBV-HCC than in EpCAM-negative HBV-HCC using Dataset GSE5975 (*p* < 0.001; Figure 1b). Since EpCAM is a biomarker of hepatic stem cells that indicates a bad prognosis [30], the findings suggest that PCLAF tv1 may play a role in the poor development of HBV-associated HCC.

To validate the bioinformatics analysis results, 42 HBV-related HCC samples and 30 non-virus-related HCC tissues were collected. PCLAF tv1 was positively stained in 81.0% (34/42) of HBV-HCC specimens and 53.3% (16/30) of non-virus HCC specimens using immunohistochemistry (IHC) (*p* < 0.05; Figure 1c,d), providing evidence that PCLAF tv1 was significantly overexpressed in HBV-HCC compared to non-virus HCC.

HepG2 cells were transiently transfected with a cloned HBV replicon pCH9/3091, which contains 1.1 copies of the HBV genome, to confirm that HBV promotes PCLAF tv1 splicing. PCLAF tv1 and tv2 were determined using specific primers, as indicated in Figure 1e. Real-time PCR revealed that the mRNA levels of PCLAF tv1 were increased by around 2.0-fold (*p* < 0.001; Figure 1f) in HepG2-pCH9/3091 cells as compared to HepG2-pCH9 cells.

A minigene construct can be used to explore alternative splicing events in vitro. The pCAS2 expression vector was utilized to generate minigene constructs with various cloning sites located between exons A and B [31]. The alternatively spliced exon 3 of PCLAF was inserted into the PCAS2 vector, as illustrated in Figure 1g. HepG2 and HepG2.2.15 cells were transfected with the minigene construct pCAS2-PCLAF Ex3. When compared to HepG2 cells, HepG2.2.15 cells yielded a 12.8-fold increase in the PCLAF tv1/tv2 ratio (*p* < 0.005; Figure 1h,i). Since HepG2.2.15 was produced by HBV DNA integration into HepG2 cells, with sustained HBV expression and replication [21], the findings suggested that HBV triggered PCLAF tv1 splicing. The pCAS2-PCLAF Ex3 minigene was also co-transfected into HepG2 cells with pCH9/3091 or a control vector. The exogenous PCLAF tv1/tv2 ratio was enhanced approximately 3.0-fold in HepG2-pCH9/3091 cells compared to HepG2-pCH9 cells (*p* < 0.001; Figure 1j,k). The findings suggested that HBV might be responsible for the abnormal inclusion of PCLAF exon 3 in HCC cells.

**Figure 2:**
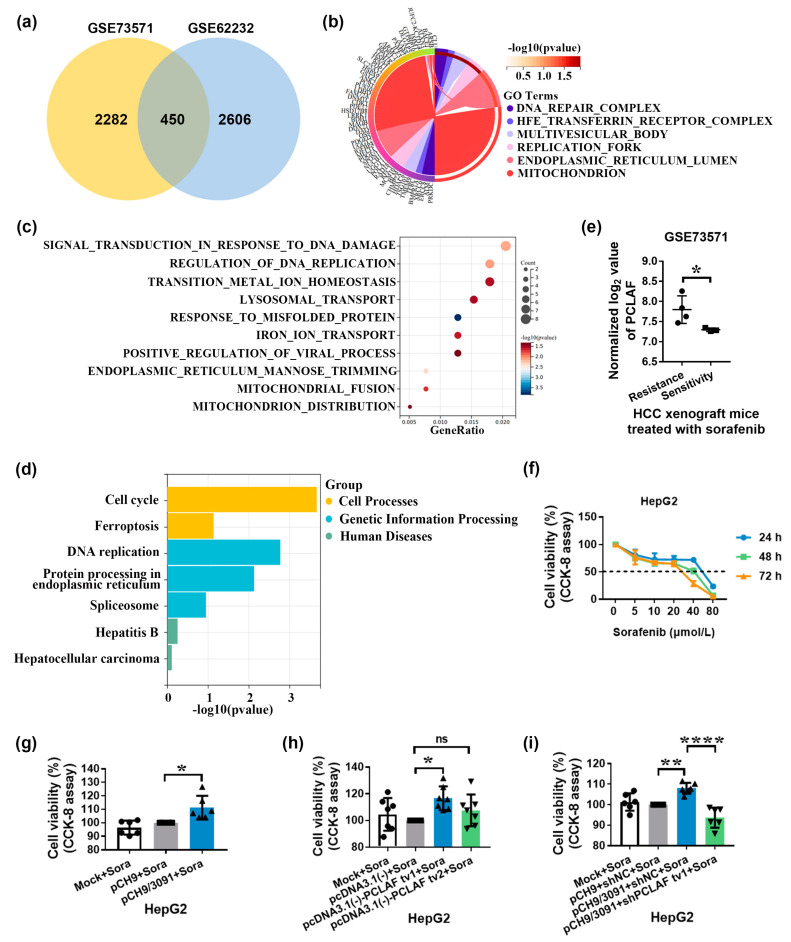
Sorafenib resistance is mediated by HBV through PCLAF tv1. The Venn diagram depicts the overlap of two datasets, GSE73571 and GSE62232 (**a**). The 450 differentially expressed genes from the Venn diagram were subjected to Gene Ontology (GO) analysis based on subcellular components (**b**) and biological processes (**c**). The 450 differentially expressed genes from the Venn diagram were classified using KEGG (**d**). The normalized log_2_ level of PCLAF in GSE73571 was shown. * *p* < 0.05 (**e**). The CCK-8 assay was used to assess the vitality of HepG2 cells after treatment with various sorafenib dosages (**f**). The CCK-8 assay was used to assess the viability of HepG2 cells transfected with pCH9 or pCH9/3091 plasmids following 30 μmol/L of sorafenib therapy. * *p* < 0.05 (**g**). The viability of HepG2 cells transfected with pcDNA3.1(-)-PCLAF tv1 or pcDNA3.1(-)-PCLAF tv2 was compared to HepG2 cells transfected with the pcDNA3.1(-) vector under 30 μmol/L of sorafenib therapy. * *p* < 0.05 (**h**). The vitality of HepG2 cells co-transfected with pCH9/3091 and shPCLAF tv1 was compared to that of HepG2 cells co-transfected with pCH9/3091 and shNC after 30 μmol/L of sorafenib treatment. ** *p* < 0.005, **** *p* < 0.0001 (**i**). “ns” not significant.

Clinical evidence suggests that HBV infection is related to a decreased response to sorafenib [32]. To discover the underlying mechanism of sorafenib resistance in HBV-related HCC, thorough analyses were performed on datasets GSE73571 (including 4 sorafenib-resistant Huh7 cell-derived xenografts and 3 Huh7 cell-derived xenografts remaining responsive to sorafenib) [33] and GSE62232 (including 16 HBV-related HCC and 37 non-virus-related HCC tissues). GSE73571 and GSE62232 both have 2732 and 3056 differentially expressed genes, respectively. The Venn diagram shows that the overlap between these two datasets includes 450 genes (Figure 2a). The common 450 differentially expressed genes were mainly enriched in cellular components such as DNA repair complex, transferrin receptor complex, replication fork, and mitochondrion (Figure 2b), which have distinct biological activities, such as transition metal ion homeostasis, iron ion transport, and viral process regulation (Figure 2c). According to KEGG enrichment analysis, the 450 differentially expressed genes were implicated in ferroptosis, spliceosome, hepatitis B, etc. (Figure 2d). Data from the GSE73571 also demonstrated that PCLAF levels in sorafenib-resistant Huh7 cell-derived xenografts were considerably higher than in sorafenib-sensitive Huh7 cell-derived xenografts (*p* < 0.05; Figure 2e), showing that PCLAF may play a role in sorafenib resistance in HBV-HCC.

To determine if HBV mediates sorafenib resistance via PCLAF, sorafenib’s cytotoxicity on HepG2 cell lines was initially tested. Sorafenib dramatically decreased the viability of HepG2 cells in a concentration-dependent manner, according to the findings (Figure 2f). Transfection of HepG2 cells with the HBV replication plasmids pCH9/3091 significantly enhanced cell viability following sorafenib treatment (*p* < 0.05), as seen in Figure 2g. The CCK-8 assay further demonstrated that PCLAF tv1 but not tv2 rescued HepG2 cells from sorafenib-induced cytotoxicity (*p* < 0.05; Figure 2h). Furthermore, knocking down PCLAF tv1 in pCH9/3091 transfected HepG2 cells drastically decreased cell viability after 48 h of sorafenib therapy (*p* < 0.0001; Figure 2i). These data suggest that HBV causes sorafenib resistance via increasing PCLAF tv1 splicing.

### 2.2. HBV Increases PCLAF tv1 Splicing by Inhibiting Trans-Acting Factor SRSF2

**Figure 3:**
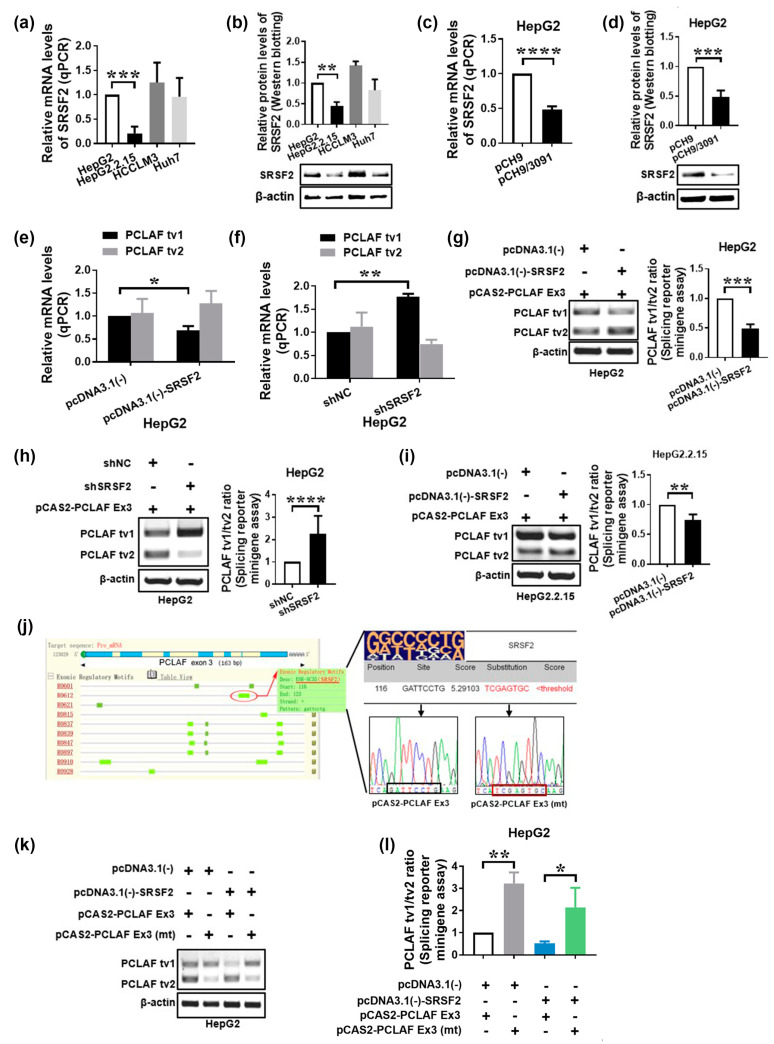
HBV suppresses SRSF2 and thus promotes PCLAF splicing. SRSF2 expression was measured by qPCR (**a**) and Western blotting (**b**) in four HCC cell lines. SRSF2 expression was determined in HepG2 cells transfected with pCH9 or pCH9/3091 using qPCR (**c**) and Western blotting (**d**). PCLAF tv1 and tv2 expression were measured in HepG2 cells with SRSF2 overexpression (**e**) or knockdown (**f**). Exogenous PCLAF tv1 and tv2 levels were measured using a splicing reporter minigene assay after transient co-transfection of the pCAS2-PCLAF Ex3 minigene and pcDNA3.1(-) or pcDNA3.1(-)-SRSF2 plasmids in HepG2 cells. The exogenous PCLAF tv1/tv2 ratio is depicted in the graph. *** *p* < 0.005 (**g**). After co-transfection of pCAS2-PCLAF Ex3 minigene with shNC or shSRSF2 plasmids, exogenous PCLAF tv1 and tv2 levels were evaluated using a splicing reporter minigene assay. The graph shows the exogenous PCLAF tv1/tv2 ratio. **** *p* < 0.0001 (**h**). Following transient co-transfection of the pCAS2-PCLAF Ex3 minigene and pcDNA3.1(-) or pcDNA3.1(-)-SRSF2 plasmids in HepG2.2.15 cells, exogenous PCLAF tv1 and tv2 levels were assessed using a splicing reporter minigene assay. The graph shows the exogenous PCLAF tv1/tv2 ratio. ** *p* < 0.005 (**i**). In silico analysis of PCLAF exon 3 using web resources ESEfinder and RegRNA. The pictogram of the degenerate motifs for SRSF2 is at the top of the table. The probable SRSF2 interaction sequences as well as the replacement sequences are depicted (**j**). Wildtype or mutant minigenes were co-transfected in HepG2 cells with either pcDNA3.1(-)-SRSF2 or pcDNA3.1(-) empty vectors. Exogenous PCLAF tv1 and tv2 expression is presented (**k**). The graph shows the exogenous PCLAF tv1/tv2 ratio. * *p* < 0.05, ** *p* < 0.005 (**l**).

We examined the expression levels of multiple SR proteins in different HCC cell lines, including HBV-replicating HCC cells, to identify the *trans*-acting mechanisms that contribute to the control of PCLAF alternative splicing in HBV-related HCC. We have found that HBV altered the expression of - SRSF2. SRSF2 mRNA (*p* < 0.001; Figure 3a) and protein levels (*p* < 0.005; Figure 3b) were significantly lower in HepG2.2.15 cells compared to HepG2. SRSF2 expression was also reduced in HepG2 cells following HBV replicon (PCH9/3091) transfection (Figure 3c,d). These findings imply that HBV reduced the expression of the *trans*-acting factor SRSF2 in HCC cells.

To learn more about the role of SRSF2 in modulating PCLAF alternative splicing, pcDNA3.1(-)-SRSF2 plasmids were transiently transfected into HepG2 cells. Overexpression of SRSF2 resulted in a significant drop in PCLAF tv1 levels (*p* < 0.05), as seen in Figure 3e. The discovery was verified by SRSF2 knockdown utilizing short-hairpin RNA (shRNA) targeted to SRSF2 (Figure 3f). In addition, the SRSF2 plasmid was co-transfected in HepG2 cells with the pCAS2-PCLAF Ex3 minigene. The exogenous PCLAF tv1/tv2 ratio was found to be considerably downregulated by SRSF2, implying that SRSF2 promotes PCLAF exon 3 skipping (*p* < 0.005; Figure 3g). The same results were verified using SRSF2 shRNA (*p* < 0.0001; Figure 3h).

Since both HBV and SRSF2 were involved in the control of PCLAF alternative splicing in HCC and HBV decreased SRSF2 expression, we postulated that HBV promoted PCLAF tv1 splicing via SRSF2. We confirmed this by transfecting the pCAS2-PCLAF Ex3 minigene into HBV-replicated HepG2.2.15 cells. Transfection of pcDNA3.1(-)-SRSF2 plasmids restored SRSF2. Although PCLAF tv1 remained the most common variation in HepG2.2.15, SRSF2 significantly reduced the exogenous PCLAF tv1/tv2 ratio in the presence of HBV (*p* < 0.005; Figure 3i). These findings suggest that HBV enhanced PCLAF tv1 splicing by inhibiting SRSF2.

PCLAF *cis*-elements that might interact with SRSF2 to control the fate of PCLAF exon 3 were predicted using in silico splicing analysis by RegRNA and ESEfinder [34,35]. Within the pCAS2-PCLAF Ex3 minigene, the predictive PCLAF *cis*-elements “*GATTCCTG*” (exon 3: 116–123) were altered to “*TCGAGTGC*”, with a binding score lower than the SRSF2 threshold predicted by ESEfinder (Figure 3j). The wildtype minigene pCAS2-PCLAF Ex3 or the mutant minigene pCAS2- PCLAF Ex3 (mt) were transfected into HepG2 cells with or without pcDNA3.1(-)-SRSF2 plasmids. The exogenous PCLAF splicing pattern was reversed following transfection with the mutant minigene, as shown in Figure 3k,l, suggesting that PCLAF exon 3 inclusion was encouraged after the *cis*-element (exon 3: 116–123) mutation. The *cis*-element “*GATTCCTG*” (PCLAF exon 3: 116–123) in PCLAF pre-mRNA was essential for SRSF2-mediated PCLAF splicing, according to these findings.

### 2.3. HBV Causes Sorafenib Resistance by Suppressing Ferroptosis via the SRSF2/PCLAF tv1 Pathway

**Figure 4:**
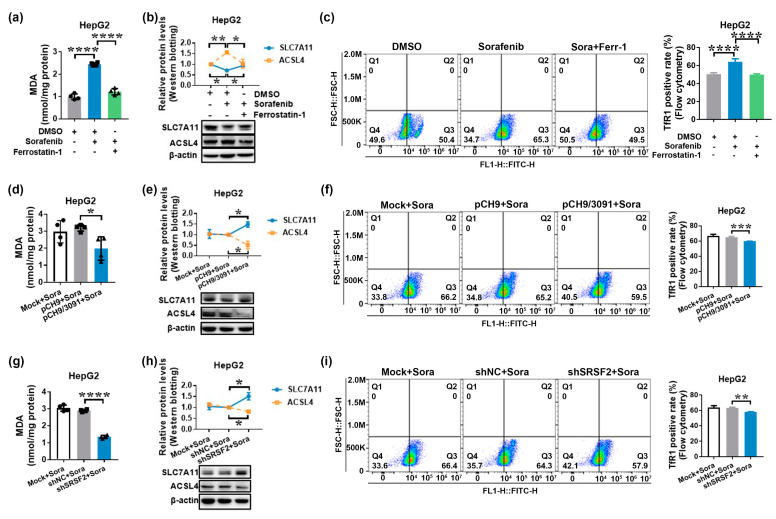
HBV and shSRSF2 prevent sorafenib-induced ferroptosis. HepG2 cells were treated for 24 h with 30 μmol/L of sorafenib with or without 10 μmol/L of ferrostatin-1. The MDA levels and expression of SLC7A11, ACSL4, and TfR1 were then determined in order to identify the involvement of sorafenib in ferroptosis in HepG2 cells (**a**–**c**). The MDA levels were determined using an MDA detection kit. The graph shows the MDA levels in HepG2 cells. **** *p* < 0.0001 (**a**). Western blotting was used to assess the expression of SLC7A11 and ACSL4. The line graph depicts the levels of SLC7A11 and ACSL4 expression in HepG2 cells. * *p* < 0.05, ** *p* < 0.005 (**b**). Flow cytometry was used to detect TfR1-positive cells. The graph displays the TfR1-positive rate. **** *p* < 0.0001 (**c**). HepG2 cells were transiently transfected with pCH9 or pCH9/3091 plasmids for 24 h before being treated for additional 24 h with 30 μmol/L of sorafenib. The MDA levels and expression of SLC7A11, ACSL4, and TfR1 were then measured to determine the role of HBV in sorafenib-induced ferroptosis in HepG2 cells (**d**–**f**). The MDA levels were determined using an MDA detection kit. The graph shows the MDA contents. * *p* < 0.05 (**d**). The SLC7A11 and ACSL4 expressions were measured by Western blotting. The line graph shows the relative expression levels of the proteins mentioned. * *p* < 0.05 (**e**). The TfR1-positive cells were detected using flow cytometry. The graph shows the TfR1-positive rate. *** *p* < 0.005 (**f**). HepG2 cells were transiently transfected with shNC or shSRSF2 plasmids for 24 h before being treated for another 24 h with 30 μmol/L of sorafenib to assess the relevance of SRSF2 in sorafenib-induced ferroptosis in HepG2 cells (**g**–**i**). An MDA detection kit was used to determine the MDA levels. The graph represents the MDA content. **** *p* < 0.0001 (**g**). Western blotting was used to assess the expression of SLC7A11 and ACSL4. The line graph illustrates the relative levels of expression of the indicated proteins. * *p* < 0.05 (**h**). The TfR1-positive cells were identified using flow cytometry. The graph shows the TfR1-positive rate. ** *p* < 0.005 (**i**). The graphs depict the mean ± SD of at least three different experiments.

Sorafenib has been shown in studies to have an anticancer effect by inducing ferroptosis in HCC [27,28]. Considering that ferroptosis is driven by iron-dependent lipid peroxidation, recognizing such lipid peroxidation events during ferroptosis is critical. As a result, one of the reactive aldehydes after lipid peroxidation malondialdehyde (MDA) was detected. In addition, specific gene expression changes can be detected, such as inhibited solute carrier family 7 member 11 (SLC7A11) or solute carrier family 3 member 2 (SLC3A2), two genes that encode the components of system X_c_^−^, and induced acyl-coenzyme A (CoA) synthetase long-chain family member 4 (ACSL4), an enzyme involved in fatty acid metabolism that is considered as a specific biomarker and driver of ferroptosis. Finally, increased transferrin receptor protein 1 (TfR1) expression promotes greater iron-loaded transferrin uptake [36,37]. As a result, at least three indicators were detected to confirm that ferroptosis occurred. As demonstrated in Figure 4a, sorafenib treatment increased MDA levels, while ferroptosis inhibitor ferrostatin-1 decreased them (*p* < 0.0001). Sorafenib decreased SLC7A11 levels while increasing ACSL4 expression. However, ferroptosis inhibitor ferrostatin-1, a lipid peroxidation inhibitor, suppressed sorafenib’s regulation of SLC7A11 and ACSL4 expression (*p* < 0.05; Figure 4b). TfR1 expression was also upregulated by sorafenib and suppressed by ferrostatin-1 therapy, as shown in Figure 4c. These findings imply that sorafenib has an anticancer impact on HCC via ferroptosis.

**Figure 5:**
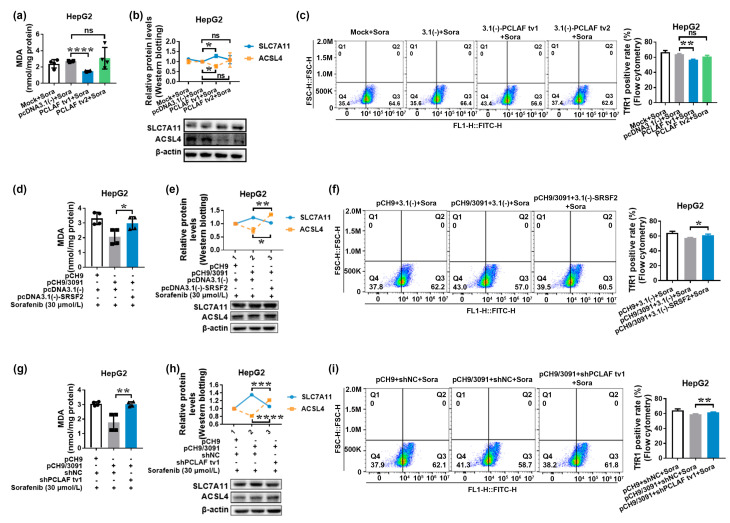
HBV protects against sorafenib-induced ferroptosis via the SRSF2/PCLAF tv1 axis. HepG2 cells were transiently transfected with pcDNA3.1(-) vector, pcDNA3.1(-)-PCLAF tv1, or pcDNA3.1(-)-PCLAF tv2 plasmids for 24 h before being treated with 30 μmol/L of sorafenib for another 24 h. The MDA levels, as well as the expression of SLC7A11, ACSL4, and TfR1, were then evaluated to determine the role of PCLAF tv1 and tv2 in sorafenib-induced ferroptosis in HepG2 cells (**a**–**c**). MDA levels were calculated using an MDA detection kit. The graph shows the MDA levels in HepG2 cells. **** *p* < 0.0001 (**a**). The expression of SLC7A11 and ACSL4 was determined by Western blotting. The line graph depicts the relative levels of SLC7A11 and ACSL4 expression in HepG2 cells. * *p* < 0.05 (**b**). TfR1-positive cells were identified by flow cytometry. The graph shows the TfR1-positive rate. ** *p* < 0.005 (**c**). HepG2 cells were transiently co-transfected with pCH9/3091 and pcDNA3.1(-) or pcDNA3.1(-)-SRSF2 plasmids for 24 h before being treated with 30 μmol/L of sorafenib for another 24 h. The ferroptosis indicators were then examined to see if HBV regulated sorafenib-induced ferroptosis in HepG2 cells via SRSF2 (**d**–**f**). An MDA detection kit was used to calculate MDA levels. The graph represents the MDA levels in HepG2 cells. * *p* < 0.05 (**d**). Western blotting was used to determine the expression of SLC7A11 and ACSL4. The line graph depicts the levels of protein expression in HepG2 cells. * *p* < 0.05, ** *p* < 0.005 (**e**). Flow cytometry was used to identify TfR1-positive cells. The graph depicts the TfR1-positive rate. * *p* < 0.05 (**f**). HepG2 cells were transiently co-transfected with pCH9/3091 and shNC or shPCLAF tv1 plasmids for 24 h before being treated for another 24 h with 30 μmol/L of sorafenib. Then, ferroptosis indicators were examined to check whether HBV regulated sorafenib-induced ferroptosis in HepG2 cells through PCLAF tv1 (**g**–**i**). MDA levels were quantified by an MDA detection kit. The graph indicates MDA contents in HepG2 cells. ** *p* < 0.005 (**g**). The expression of SLC7A11 and ACSL4 was determined by Western blotting. The line graph shows the relative protein expression levels. *** *p* < 0.005, **** *p* < 0.0001 (**h**). Flow cytometry was used to identify TfR1-positive cells. The graph illustrates the TfR1-positive rate. ** *p* < 0.005 (**i**). The graphs show the mean ± SD of at least three separate tests. “ns” not significant.

We hypothesized that PCLAF would participate in HBV-mediated sorafenib resistance by inhibiting ferroptosis based on the findings that overlapping differentially expressed genes between sorafenib-resistant HCC and HBV-related HCC were implicated in ferroptosis. Figure 4d–f demonstrate that pCH9/3091 overexpression increased MDA levels, increased SLC7A11 levels, and decreased ACSL4 and TfR1 levels following sorafenib treatment, indicating that HBV suppressed sorafenib-induced ferroptosis in HepG2 cells. Furthermore, the knockdown of SRSF2 (Figure 4g–i) and overexpression of PCLAF tv1 but not tv2 (Figure 5a–c) play a similar role to HBV in regulating MDA levels, SLC7A11, ACSL4, and TfR1 expression levels, suggesting that HBV, knockdown of SRSF2, and overexpression of PCLAF tv1 all contribute to inhibition of sorafenib-induced ferroptosis.

Furthermore, supplementing SRSF2 expression in HepG2 cells with pcDNA3.1(-)-SRSF2 elevated MDA levels, which were lowered by pCH9/3091 following sorafenib treatment (Figure 5d). When pcDNA3.1(-)-SRSF2 and pCH9/3091 co-transfected HepG2 cells were compared to pcDNA3.1(-) and pCH9/3091 co-transfected HepG2 cells after sorafenib treatment, SLC7A11 was downregulated, whereas ACSL4 and TfR1 were upregulated (Figure 5e,f). According to the findings, HBV suppressed sorafenib-induced ferroptosis by inhibiting SRSF2. Similarly, co-transfected HepG2 cells with shPCLAF tv1 and pCH9/3091 raised MDA levels, ACSL4, and TfR1 expression, but decreased SLC7A11 expression after sorafenib therapy compared to shNC and pCH9/3091 co-transfected HepG2 cells (Figure 5g–i). The findings suggest that HBV suppressed sorafenib-induced ferroptosis via increased PCLAF tv1 splicing. As a result, the facts presented above demonstrate that HBV prevents sorafenib-induced ferroptosis via the SRSF2/PCLAF tv1 axis.

### 2.4. HBV Suppresses Sorafenib-Induced Ferroptosis through SRSF2/PCLAF tv1 via Both the Fe^2+^ and Glutathione Peroxidase 4 (GPX4) Pathways

**Figure 6:**
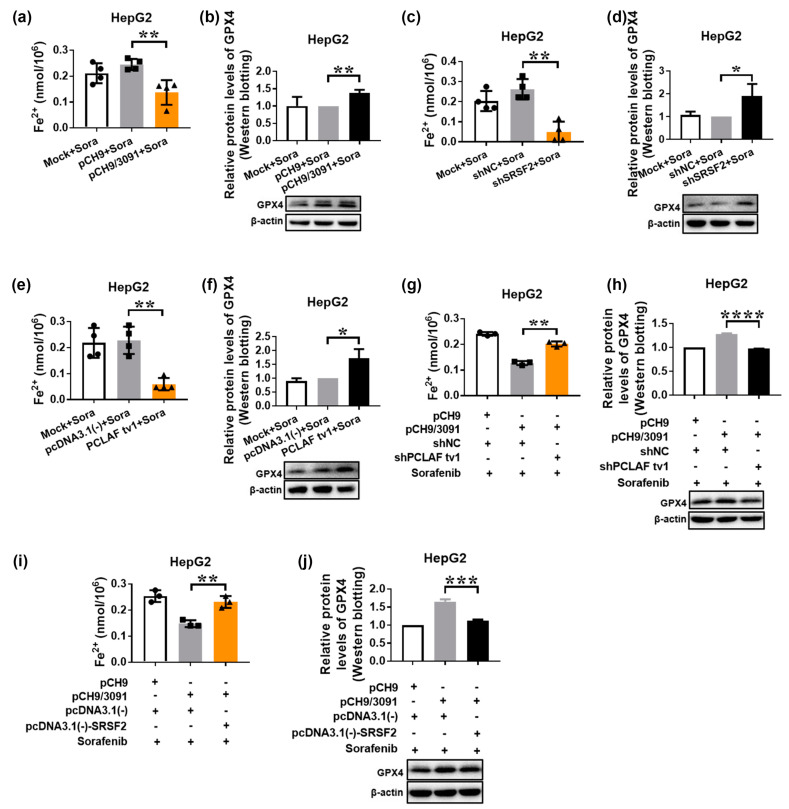
HBV suppresses intracellular Fe^2+^ levels and induces GPX4 expression to prevent sorafenib-induced ferroptosis via the SRSF2/PCLAF tv1 pathway. HepG2 cells were transiently transfected with the indicated plasmids for 24 h before being treated with 30 μmol/L of sorafenib for another 24 h. The Cell Ferrous Iron Colorimetric Assay Kit was used to determine the intracellular ferrous iron level (**a**,**c**,**e**,**g**,**i**). Western blotting was used to assess GPX4 expression (**b**,**d**,**f**,**h**,**j**). The graph shows the intracellular Fe^2+^ levels after sorafenib treatment in pCH9 or pCH9/3091 transfected HepG2 cells. ** *p* < 0.005 (**a**). After sorafenib treatment, GPX4 protein levels were determined in pCH9 or pCH9/3091 transfected HepG2 cells. ** *p* < 0.005 (**b**). The graph shows the intracellular Fe^2+^ levels after sorafenib therapy in shNC or shSRSF2 transfected HepG2 cells. ** *p* < 0.005 (**c**). GPX4 protein levels were measured in shNC or shSRSF2 transfected HepG2 cells after sorafenib treatment. * *p* < 0.05 (**d**). The graph represents the intracellular Fe^2+^ levels in HepG2 cells transfected with pcDNA3.1(-) or pcDNA3.1(-)-PCLAF tv1 following sorafenib treatment. ** *p* < 0.005 (**e**). After sorafenib therapy, GPX4 protein levels were tested in pcDNA3.1(-) or pcDNA3.1(-)-PCLAF tv1 transfected HepG2 cells. * *p* < 0.05 (**f**). The graph indicates the intracellular Fe^2+^ levels in HepG2 cells co-transfected with pCH9/3091 and shNC or shPCLAF tv1 after sorafenib treatment. ** *p* < 0.005 (**g**). After sorafenib stimulation, GPX4 protein levels were measured in pCH9/3091 and shNC or shPCLAF tv1 co-transfected HepG2 cells. **** *p* < 0.0001 (**h**). The graph shows the intracellular Fe^2+^ levels in HepG2 cells co-transfected with pCH9/3091 and pcDNA3.1(-) or pcDNA3.1(-)-SRSF2 after sorafenib treatment. ** *p* < 0.005 (**i**). GPX4 protein levels were determined in pcDNA3.1(-) or pcDNA3.1(-)-SRSF2 co-transfected HepG2 cells after sorafenib treatment. *** *p* < 0.005 (**j**). All graphs depict the mean ± SD of at least three different tests.

The iron metabolism upregulated intracellular free Fe^2+^ level may directly generate excessive reactive oxygen species (ROS) through the Fenton reaction, hence promoting lipid peroxidation [26]. By decreasing phospholipid hydroperoxide formation to the equivalent nonfatal phospholipid alcohol, GPX4 as a phospholipid hydroperoxidase efficiently removes ROS and suppresses lipid peroxidation [38]. As a result of the equilibrium of these two mechanisms, ferroptosis is effectively controlled. Intracellular free Fe^2+^ levels and GPX4 protein levels were measured to establish which route was involved in HBV-suppressed sorafenib-induced ferroptosis. Figure 6a,b indicate that pCH9/3091 transfection significantly reduced intracellular free Fe^2+^ levels while increasing GPX4 protein levels following sorafenib treatment, demonstrating that HBV suppresses sorafenib-induced ferroptosis by both eliminating the Fe^2+^ route and activating the GPX4 pathway. Furthermore, shSRSF2 (Figure 6c,d) or pcDNA3.1(-)-PCLAF tv1 (Figure 6e,f) produced similar results to pCH9/3091 in terms of intracellular free Fe^2+^ levels and GPX4 protein levels, indicating that SRSF2 knockdown and PCLAF tv1 overexpression both inhibit sorafenib-induced ferroptosis via both pathways.

Furthermore, we co-transfected HepG2 cells with pCH9/3091 and shNC or shPCLAF tv1 plasmids, followed by 48 h of sorafenib treatment. When pCH9/3091 and shPCLAF tv1 co-transfected HepG2 cells were compared to the control, the intracellular free Fe^2+^ level increased while the GPX4 protein level decreased. The findings indicate that HBV inhibits the Fe^2+^ route while boosting the GPX4 pathway through PCLAF tv1 (Figure 6g,h). Similarly, pCH9/3091 and pcDNA3.1(-)-SRSF2 co-transfected HepG2 cells increased intracellular free Fe^2+^ levels while decreasing GPX4 protein levels after sorafenib treatment (Figure 6i,j). These findings strongly suggest that HBV suppresses sorafenib-induced ferroptosis via the SRSF2/PCLAF tv1 pathway by blocking the Fe^2+^ pathway and activating the GPX4 pathway.

### 2.5. The SRSF2/PCLAF tv1 Axis Is Engaged in HBV-Mediated Sorafenib Resistance by Suppressing Ferroptosis

**Figure 7:**
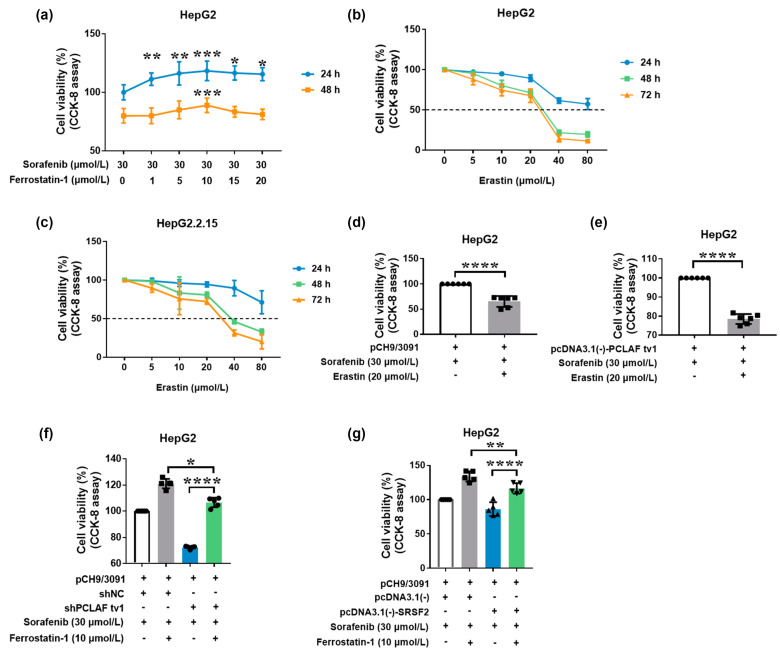
HBV causes sorafenib resistance by suppressing ferroptosis via the SRSF2/PCLAF tv1 axis. HepG2 cells were treated with a variety of ferrostatin-1 concentrations in the presence of 30 μmol/L of sorafenib. The cell viability was determined using the CCK-8 assay (**a**). The CCK-8 assay was used to determine the cell viability following treatment with various erastin doses in HepG2 (**b**) and HepG2.2.15 cells (**c**). HepG2 cells transiently transfected with pCH9/3091 were treated for 24 h with or without 20 μmol/L of erastin in the presence of 30 μmol/L of sorafenib. CCK-8 was then employed to assess cell viability (**d**). HepG2 cells transiently transfected with pcDNA3.1(-)-PCLAF tv1 were treated for 24 h with or without 20 μmol/L of erastin in the presence of 30 μmol/L of sorafenib. Then, the cell viability was assessed using CCK-8 (**e**). Transiently co-transfected HepG2 cells with pCH9/3091 and shNC or shPCLAF tv1 were then treated for 24 h with or without 10 μmol/L of ferrostatin-1 in the presence of 30 μmol/L of sorafenib. CCK-8 was employed to assess cell viability (**f**). HepG2 cells were transiently co-transfected with pCH9/3091 and pcDNA3.1(-) or pcDMA3.1(-)-SRSF2, then treated for 24 h with or without 10 μmol/L of ferrostatin-1 in the presence of 30 μmol/L of sorafenib. CCK-8 was used to test cell viability (**g**). All graphs display the mean ± SD of at least three separate tests. * *p* < 0.05, ** *p* < 0.005, *** *p* < 0.005, **** *p* < 0.0001.

To investigate the function of ferroptosis in sorafenib resistance, HepG2 cells were treated with a variety of doses of the ferroptosis inhibitor ferrostatin-1. The results showed that ferrostatin-1 reduced the cytotoxic effects of sorafenib on HepG2 cells (Figure 7a), implying that ferroptosis may contribute to HepG2 cell sensitivity to sorafenib.

Erastin, a ferroptosis agonist [39], on the other hand, was also employed to treat HCC cells. Erastin increased cell mortality in both HepG2 and HepG2.2.15 cells in a concentration-dependent manner, as seen in Figure 7b,c. However, when treated with erastin at the same concentration, HepG2.2.15 viability was higher than HepG2. The findings imply that HBV may impede erastin-induced ferroptosis. HepG2 cells transfected with pCH9/3091 plasmids were treated with or without erastin in the presence of sorafenib to further investigate the function of ferroptosis in HBV-induced sorafenib resistance. The treatment with erastin dramatically reduced cell viability (*p* < 0.0001; Figure 7d), indicating that ferroptosis can ameliorate HBV-induced sorafenib resistance.

In addition, the role of ferroptosis in PCLAF tv1-mediated sorafenib resistance was explored. Erastin treatment significantly reduced cell viability in pcNDA3.1(-)-PCLAF tv1 overexpressed HepG2 cells after sorafenib treatment (*p* < 0.0001; Figure 7e). The results showed that ferroptosis can boost the sensitivity of PCLAF tv1 overexpressed HepG2 cells to sorafenib.

We then investigated whether HBV decreased HepG2 cell susceptibility to sorafenib by suppressing ferroptosis through PCLAF tv1. pCH9/3091 and shNC or shPCLAF tv1 were co-transfected into HepG2 cells, which were then treated with 30 μmol/L of sorafenib with or without 10 μmol/L of ferrostatin-1. The CCK-8 assay revealed that when ferroptosis was inhibited by ferrostatin-1, the knockdown of PCLAF tv1 dramatically reduced cell viability relative to the shNC group. Furthermore, after PCLAF tv1 knockdown, ferrostatin-1-treated HepG2 cells had better cell viability than HepG2 cells that had not been treated with ferrostatin-1 (Figure 7f). The findings suggest that HBV induced HepG2 cell tolerance to sorafenib via suppressing ferroptosis through PCLAF tv1.

To investigate the function of ferroptosis in HBV-mediated sorafenib resistance by SRSF2, pCH9/3091 and pcDNA3.1(-) or pcDNA3.1(-)-SRSF2 were co-transfected into HepG2 cells with or without sorafenib. The CCK-8 assay revealed that SRSF2 overexpression dramatically reduced cell viability following ferrostatin-1 treatment when compared to the control. Furthermore, when SRSF2 was overexpressed, ferrostatin-1-treated HepG2 cells had better cell viability than HepG2 cells that had not been treated with ferrostatin-1 (Figure 7g). The findings suggest that HBV generated HepG2 cell tolerance to sorafenib by suppressing ferroptosis via SRSF2.

**Figure 8:**
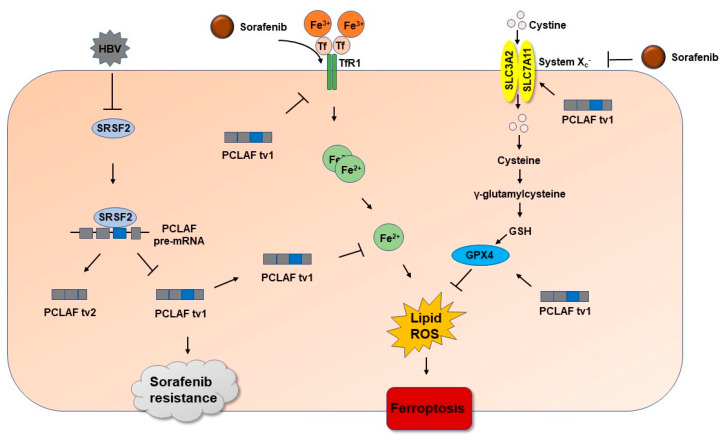
The diagram demonstrates the pathway whereby HBV stimulates PCLAF tv1 splicing by decreasing SRSF2 and contributes to sorafenib resistance in HCC by preventing ferroptosis. HBV promotes PCLAF tv1 splicing by inhibiting SRSF2. Sorafenib therapy induces TfR1-Fe^2+^-induced ferroptosis while suppressing GPX4-suppressed ferroptosis in HCC. HBV inhibits ferroptosis via the SRSF2/PCLAF tv1 pathway, which acts on both the TfR1-Fe^2+^ and the GPX4 pathways and hence mediates sorafenib resistance.

As a result, the above findings demonstrated that HBV plays an essential role in sorafenib resistance by suppressing ferroptosis through the SRSF2/PCLAF tv1 axis (Figure 8).

## 3. Discussion

HCC is the leading cause of cancer death worldwide [1]. When patients are discovered at an advanced stage, the prognosis remains poor, owing primarily to chemoresistance [40]. Many factors have been documented to contribute to chemoresistance in HCC to date. HBV is one of the most important, causing chemoresistance in HCC. Chemotherapy clinical trials have shown that HBsAg-positive HCC patients had a lower survival benefit [32,41]. On the other hand, anti-apoptotic isoforms produced by RNA splicing may confer resistance to chemotherapy [42]. Our previous study has shown that CKLF1, variant 2 of the chemokine-like factor, and PCLAF tv1 contributed to doxorubicin resistance in HCC cells [17,43]. Furthermore, reducing ferroptosis may contribute to sorafenib resistance in HCC [44]. However, no research has been conducted to investigate the function of ferroptosis and alternative splicing in HBV-induced sorafenib resistance. Our findings reveal that HBV induced PCLAF tv1 splicing in HCC cells by inhibiting the *trans*-acting protein SRSF2. HBV also reduced sorafenib-induced ferroptosis via the SRSF2/PCLAF tv1 axis. In contrast, reducing ferroptosis prevented HBV-mediated sorafenib resistance caused by the SRSF2/PCLAF tv1 pathway.

PCLAF, also known as KIAA0101, PAF, and HCV NS5A-transactivated protein 9 (NS5ATP9), was initially identified as a proliferating cell nuclear antigen (PCNA)-related protein [45]. PCLAF overexpression was more likely in HCC patients with serum HBsAg positivity in Yuan et al.’s study [46]. In this investigation, we discovered that PCLAF tv1 expression was high in HBV-associated HCC tissues and HBV-replicating cell lines, indicating that PCLAF tv1 may be a critical factor in HBV-induced HCC. Interestingly, PCLAF is overexpressed in late-stage HCC and is linked to a low overall survival rate [46,47]. PCLAF probably plays a role in chemoresistance. Indeed, PCLAF is involved in cisplatin resistance in ovarian cancer [48] and esophageal cancer [49]. Our previous study showed that PCLAF tv1 contributed to doxorubicin resistance in HCC [17]. In this study, we also observed that PCLAF tv1 was involved in HBV-induced sorafenib resistance, but not tv2.

With almost 90% of human genes undergoing alternative splicing, alternative splicing is postulated as a critical method for expanding proteome diversity [50]. In the early 1990s, SR family members were first reported as splicing factors [51] to promote exon inclusion by interacting with specific sequence elements in exons, known as exonic splicing enhancers (ESEs) [9,52]. However, evidence suggests that SR family proteins also inhibit cassette exon inclusion [23,53]. SRSF2 has been shown to activate both exon inclusion and exon skipping events [54]. SRSF2 may play multiple functions in the formation of HCC. SRSF2 levels in HCC tissues have been found to be higher [24,55]. By modulating the production of oncogenic mutations, SRSF2 modulates cell proliferation and tumorigenic potential [24]. Another study found that deleting SRSF2 causes hepatic progenitor cell regeneration, activation of oncofetal genes, and numerous signaling pathways, ultimately leading to HCC formation in mice [56]. In this study, we revealed that SRSF2 induced exon 3 skipping in both endogenous and exogenous PCLAF in HCC cells. PCLAF Ex3 (116–123) “*GATTCCTG*” was identified as a potential binding site for SRSF2, which is required for PCLAF pre-mRNA exon 3 skipping. In liver cancer, HBV has been shown to increase the splicing of oncogenic variants. HBV core protein may inhibit the expression of the proapoptotic form of Fas while increasing the expression of the anti-apoptotic variant [19]. Exon 4 of the sterile alpha motif domain and HD domain-containing protein 1 (SAMHD1), a predictor of HCC occurrence, was often inserted in HBV-positive HCC [20]. Our findings also demonstrated that HBV triggered PCLAF tv1 splicing in HCC cells through downregulating SRSF2.

Sorafenib has been shown to cause ferroptosis. Failure of ferroptosis, on the other hand, can cause inflammation-associated immunosuppression in the tumor microenvironment, encouraging tumor growth [57]. HBV decreased the occurrence of ferroptosis, according to accumulating evidence. Exosomal miR-222 released by HBV-infected hepatocytes may target transferrin receptor TfR1 to reduce the iron burden and prevent ferroptosis in hepatic stellate cells [58]. Exogenous HBx expression in hepatic stellate cells increases the expression of SLC7A11 and GPX4 proteins, preventing cell death via ferroptosis [59]. In Haga et al.’s study, sorafenib therapy increased the expression and phosphorylation of c-Jun in human hepatoma cell lines with HBV integration, such as PLC/PRF/5 and HepG2.2.15. Sorafenib resistance was linked to c-Jun overexpression in HCC cells [60]. Overexpression of c-Jun combined with simultaneous modification of c-Jun proteins by O-linked-N-acetyl-glucosamine inhibited ferroptosis in human HCC cells via increased GSH synthesis [61], implying that HBV may prevent sorafenib-induced ferroptosis in HCC cells via increased specific modified c-Jun. Here, we found that HBV suppressed sorafenib-induced ferroptosis in HCC cells through the SRSF2/PCLAF tv1 pathway by lowering intracellular Fe^2+^ levels and boosting GPX4 expression.

Many studies have found that inhibiting ferroptosis facilitates sorafenib resistance [44,62,63]. A ferroptosis inducer, such as erastin, improves hepatoma cell sensitivity to sorafenib [64]. The ferroptosis inducer erastin improved sorafenib sensitivity in both HBV-positive and PCLAF tv1 overexpressed HepG2 cells, according to our findings. HBV also has a role in sorafenib resistance by suppressing ferroptosis via the SRSF2/PCLAF tv1 axis. Consequently, we hypothesized that inhibiting SRSF2 expression by HBV facilitated PCLAF tv1 splicing, which contributed to sorafenib resistance by reducing ferroptosis.

## 4. Materials and Methods

### 4.1. Bioinformatics Analysis

The Gene Expression Omnibus (GEO) dataset GSE62232 [29], which contains 16 cases of HCC specimens with HBV infection and 37 cases of non-virus HCC specimens, was searched for differentially expressed genes in HBV-related HCC. The platform for this work was GPL570 (Human Genome U133 Plus 2.0 Array, Affymetrix, Santa Clare, CA, USA).

PCLAF expression was examined in the dataset GSE5975 [30], which contained 94 EpCAM-positive HBV-positive HCC and 143 EpCAM-negative HBV-positive HCC tissues. The platform for this work was GPL1528 (NCI/ATC Hs-OperonV2). Dataset GSE73571 [33], which included 4 sorafenib-resistant Huh7 cell-derived xenografts and 3 Huh7 cell-derived xenografts that remained susceptible to sorafenib, was searched for genes that were significantly dysregulated in sorafenib-resistant HCC cells. The platform for this work was GPL6244 (Human Gene 1.0 ST Array, Affymetrix, Santa Clare, CA, USA). For bioinformatics analysis, the Limma package [65] of R software was used with a *p*-value < 0.05 as the standard. All data extracted from bioinformatics databases were presented as log_2_ value.

To demonstrate the activities of target genes in cellular components and biological processes, Gene Ontology (GO) annotation was performed on R software target genes. Pathway analysis was performed using the Kyoto Encyclopedia of Genes and Genomes (KEGG) to identify significant pathways associated with target genes. BioVenn, a web application [66], was used to create the Venn diagram.

KIAA0101 exon 3 was analyzed in silico utilizing the web resources ESEfinder and RegRNA [34,35]. Only putative sites having a score of more than 5, exceeding the threshold value (2.38 for SRSF2), were examined for ESEfinder

### 4.2. Clinical Tissues

HCC tissues were acquired from patients who had surgery at Wuhan University’s Renmin Hospital (Wuhan, China). The patients were divided into two groups based on the presence of viral hepatitis serological markers: the HBV-related HCC group, which included 42 patients who were positive for HBsAg but negative for anti-HCV, and the non-virus-related HCC group, which included 30 patients who were negative for both HBsAg and anti-HCV. The sample collection was carried out in accordance with consensus agreements and was approved by the ethics committee of Wuhan University, School of Medicine (Ethics No. 14011). The study followed the International Ethical Guidelines for Biomedical Research Involving Human Subjects (CIOMS).

### 4.3. IHC

Here, 4% paraformaldehyde was used to fix the clinical samples. After that, the samples were fixed in paraffin and sectioned. The primary polyclonal antibody (sc-65163, Santa Cruz Biotechnology, CA, USA) was used to stain PCLAF tv1, followed by peroxidase-conjugated rabbit anti-goat IgG (sc-2768, Santa Cruz Biotechnology, CA, USA). The intensity of PCLAF tv1 staining was graded from 0 to 3 as follows: A score of 0 indicates that no membranous staining is present in any of the tumor cells, a score of 1 indicates that there is staining in less than 10% of the tumor cells with any intensity or less than 30% of the tumor cells with weak intensity, a score of 2 indicates that there is staining in 10–30% of the tumor cells with moderate-to-strong intensity or staining in 30–50% of the tumor cells with weak-to-moderate intensity, and a score of 3 indicates that there is staining in more than 50%. Positive samples received a score of 2 or above.

### 4.4. Plasmids’ Construction

Plasmid pcDNA3.1-SRSF2-cMyc was from Dr. Kathleen Scotto (Addgene plasmid 44721). We amplified the full-length SRSF2 gene and cloned it into the pcDNA3.1(-) vectors, as directed by the manufacturer (V795-20, Invitrogen, Burlington, ON, USA). PCLAF tv1 and SRSF2 were knocked down using shRNA. PCLAF tv1 was targeted by the sequence 5′-UCAGAUUCCUGAAGAGGCA-3′. The target sequence for SRSF2 was 5′-UUGUGUAGCAGUUGAGUAAUGCUGGUUAG-3′. The shRNA-coding oligonucleotides were annealed and ligated into the BamHI/HindIII restriction site of pSilencer 2.1-U6 neo vector (AM5764, Ambion, Austin, TX, USA). Control pSilencer 2.1-U6 neo vector contained a scrambled sequence: 5′-TCTTAATCGCGTATAAGGC-3′.

DNA fragments including PCLAF wildtype exon 3 and surrounding intronic sequences were amplified from the genomic DNA of HepG2 cells using the primers Forward 1 and Reverse 1. The PCR products were inserted into the intron of the pCAS2 vector containing a two-exon splicing reporter minigene to generate pCAS2-PCLAF Ex3. Plasmid pCAS2 was provided by Dr. Alexandra Martins (University of Rouen, Rouen, France).

Minigenes pCAS2-PCLAF Ex3 (mt) carrying the mutant PCLAF exon 3 variant were prepared by site-directed mutagenesis through the two-stage overlap extension PCR method by using primers Forward 2, Reverse 2, Forward 3, and Reverse 3.

Supplementary Table S1 lists the primer sequences. All constructs were confirmed by sequencing.

### 4.5. Cell Culture and Transient Transfection

Human hepatoma cell lines HepG2, HepG2.2.15, HCCLM3, and Huh7 were purchased from American Type Culture Collection (ATCC, Manassas, VA, USA). All cell lines were cultured at 37 ℃ in 5% CO_2_ in Dulbecco’s modified Eagle’s medium (2347432, GIBCO BRL, CA, USA), with 10% fetal bovine serum, 100 units/mL of penicillin, and 100 µg/mL of streptomycin sulfate. Lipofectamine 2000 (11668019, Invitrogen; Carlsbad, CA, USA) was used to transiently transfect cells according to the manufacturer’s procedure.

### 4.6. RNA Extraction and Real-Time PCR

Total RNA was extracted using TRIzol^®^ reagent (15596026, Invitrogen, Carlsbad, CA, USA) according to the manufacturer’s instructions. After DNase I (EN0521, Thermo Fisher Scientific, Markham, ON, Canada) was used to remove genomic DNA contaminations from total RNA, 1 μg of RNA was converted into cDNA using ReverTra Ace (FSK-101, TOYOBO, Osaka, Japan) according to the manufacturer’s procedure.

Real-Time PCR was performed on the iCycler System (C1000, Bio-Rad, Hercules, USA). Comparative quantification was used, with PCLAF tv1, PCLAF tv2, and SRSF2 normalized to an internal control gene (β-actin). The primer sequences are listed in Supplementary Table S1. The reactions were incubated at 95 °C for 10 min, then 40 cycles of 95 °C for 10 s, 58 °C for 10 s, 72 °C for 10 s, and 60 °C for 1 min. The results were presented as 2(−ΔΔCt) values.

### 4.7. Splicing Reporter Minigene Assay

The relevant cell lines were transiently transfected with wildtype or mutant minigene constructs. Total RNA was extracted 48 h after transfection. Denaturation at 94 °C for 5 min, 22 cycles of amplification with denaturation at 94 °C for 30 s, annealing at 56 °C for 30 s, and extension at 72 °C for 30 s, and a final extension at 72 °C for 10 min were performed in a Bio-Rad thermocycler. On 2% EtBr-stained agarose gels, PCR products were detected, and band intensity was quantified using the Bioprofil (Bio-1D) equipment (Vilber Lourmat, France). The transcriptional study of pCAS2-KIAA0101 and pCAS2-KIAA0101mt minigenes was carried out using Forward 4 and Reverse 4 primers (Supplementary Table S1).

### 4.8. Drug Treatment

Sorafenib (IS0220, Solarbio, Beijing, China), ferrostatin-1 (#HY-100579, MCE, NJ, USA), and erastin (IE0310, Solarbio, Beijing, China) were diluted to a concentration of 20 mmol/L in dimethyl sulfoxide (DMSO). Sorafenib was added to the culture media either directly or after plasmid transfection in serial dilutions or at the given concentrations. The CCK-8 assay was used to determine the IC_50_ of the cells after they had been grown for 1–3 days. The cells were grown for 24 h before being evaluated for other markers.

### 4.9. CCK-8 Assay

The cell viability was determined using CCK-8 assay kits (Dojindo Laboratories, Kumamoto, Japan). Cells were plated in a 96-well cell culture plate at a density of 5 × 10^3^ cells per well and cultured in growth media for 24 h before being transiently transfected with plasmids. After another 48 h at 37 °C, the cells were rinsed with PBS solution. The medium was changed for a new serum-supplemented medium. The cells were then treated for 40 min with the CCK test solution at the specified dose. A Multiskan FC plate reader was used to measure the absorbance at 450 nm, which was then analyzed using Skanlt for Multiscan FC software (Thermo Scientific, Waltham, MA, USA).

### 4.10. Protein Isolation and Western Blotting Analysis

The cells were cleaned with 1 × PBS before being lysed with M-PER mammalian protein extraction reagent (78501, Thermo Fisher Scientific, Waltham, MA, USA), supplemented with a Cocktail (11873580001, Roche, Basle, Switzerland). Protein concentrations were measured as indicated by the manufacturer using the BCA protein assay kit (23250, Thermo Fisher Scientific, Waltham, MA, USA).

Protein samples (30 μg) were separated in SDS-PAGE gels ranging from 4% to 15% and transferred to nitrocellulose membranes (Amersham Pharmacia Biotech, San Francisco, CA, USA). After blocking in 5% skim milk for 60 min, the membranes were treated with the appropriate primary antibodies and fluorescent-conjugated secondary antibodies. Primary antibodies against SRSF2 (ab204916, 1:1000, Abcam, Cambridge, UK), SLC7A11 (A2413, 1:1000, ABclonal, Wuhan, China), ACSL4 (A6826, 1:1000, ABclonal, Wuhan, China), and GPX4 (A1933, 1:1000, ABclonal, Wuhan, China) were used. The internal standard was probed with an anti-β-actin-peroxidase monoclonal antibody (A3854, Sigma-Aldrich, Bedford, MA, USA). In a Tanon 5200 MultiImage System (Tanon Science & Technology, Shanghai, China), ECL reagents (34080, Millipore, Burlington, MA, USA) were used to observe the bands. ImageJ software (National Institutes of Health, Bethesda, MD, USA) was used to calculate band intensities.

### 4.11. Lipid Peroxidation Assessment

The level of thiobarbituric acid reactive substance (TBARS) was evaluated using the Lipid Peroxidation MDA Assay Kit (S0131S, Beyotime Biotechnology, Shanghai, China). Cells were planted in 6 mm plates at a density of 2 × 10^6^ cells per plate. After 24 h of drug treatment, cells were washed with ice-cold 1 × PBS, lysed with RIPA on ice, and centrifuged at 15,000× *g* for 10 min to remove insoluble material. The BCA protein assay kit (23250, Thermo Fisher Scientific, Waltham, MA, USA) was used to determine the protein content. The supernatant (100 μL) was incubated for 15 min at 100 °C with 200 μL of MDA test detection solution before being cooled to room temperature. The supernatant was collected by centrifuging the mixture at 1000× *g* for 10 min. Then, 200 μL of supernatant was transferred to a 96-well plate, and absorbance at 532 nm was recorded using a Multiskan FC plate reader and analyzed using Skanlt for Multiscan FC software (Thermo Scientific, Waltham, MA, USA). The TBARS concentration was estimated in nmoL/mg protein.

### 4.12. Flow Cytometry

Flow cytometry was used to identify TfR1-positive cells. A total of 1 × 10^6^ cells were collected and washed in ice-cold 1 × PBS. For 30 min on ice, the cells were resuspended in 5% BSA. The cells were treated for 1 h on ice with 0.2 g per 10^6^ cells with ABfloTM 488 rabbit anti-human CD71 mAb (A22301, ABclonal, Wuhan, China). The cells were centrifuged three times in 5 min with 1 × PBS. A total of 10,000 cells per sample were examined for fluorescence intensity on the FL1 channel using an Epics Altra II cytometer (Beckman Coulter, Miami, FL, USA) with gating to record only living cells (gate constructed from the non-antibody treatment group). FlowJo software (Tree star, Inc., Ashland, OR, USA) was used for the analysis.

### 4.13. Cell Ferrous Iron Colorimetric Assay

The Cell Ferrous Iron Colorimetric Assay Kit (E-BC-K881-M, Elabscience, Wuhan, China) was used to determine the intracellular ferrous iron level. Approximately 1 × 10^6^ cells were extracted and homogenized on ice for 10 min with 200 μL of lysis buffer before being centrifuged at 15,000× *g* for 10 min to collect the supernatant. Then, 80 μL of supernatant was treated with the iron probe or the control reagent for 10 min at 37 °C. A Multiscan FC plate reader was used to measure the absorbance at 593 nm, which was then analyzed using Skanlt for Multiscan FC software (Thermo Scientific, Waltham, MA, USA). The relative ferrous iron level equals the difference between the experimental and control groups’ ferrous iron contents. A cell ferrous iron standard curve was used to calculate the cell ferrous iron content.

### 4.14. Statistical Analysis

The Chi-squared or Fisher’s exact tests were employed to compare qualitative variables. To express numerical data, the mean ± standard deviation (SD) or mean ± standard error of the mean (SEM) of at least three independent experiments was utilized. To compare significant differences between treatments, an unpaired Student’s t-test, one-way ANOVA followed by Fisher’s post hoc comparison test, or two-way ANOVA with multiple post hoc comparisons were used. The graphical representations were created using the GraphPad Prism software (GraphPad, San Diego, CA, USA). Statistical significance was represented by *p* < 0.05 (*), *p* < 0.005 (**), *p* < 0.001 (***), and *p* < 0.0001 (****). At least three distinct trials were used in the research.

## 5. Conclusions

In conclusion, our data suggest that HBV promotes sorafenib resistance in HCC by lowering SRSF2 and reducing ferroptosis via deregulation of PCLAF tv1 alternative splicing. Our findings identified a potential molecular therapeutic target in addition to a predictor of sorafenib resistance in HBV-related HCC. The findings also point to a possible target for combination therapy by triggering ferroptosis in HBV-related HCC to increase sorafenib resistance.

## Data Availability

The data presented in this study are available in this article.

## Supplementary Materials

The following supporting information can be downloaded at: https://www.mdpi.com/article/10.3390/ijms24043263/s1.
